# Supplementary material for: Extraordinary pseudocapacitive energy storage triggered by phase transformation in hierarchical vanadium oxides
Source: Nat Commun. 2018 Apr 10;9:1375. doi: 10.1038/s41467-018-03700-3 (PMC5893573; doi:10.1038/s41467-018-03700-3)
Supplement: Supplementary file 1 — Supplementary Information(PDF 4585 kb) [file 41467_2018_3700_MOESM1_ESM.pdf]

# Supplementary information

# Extraordinary pseudocapacitive energy storage triggered by phase transformation in hierarchical vanadium oxides

**Liu et al**

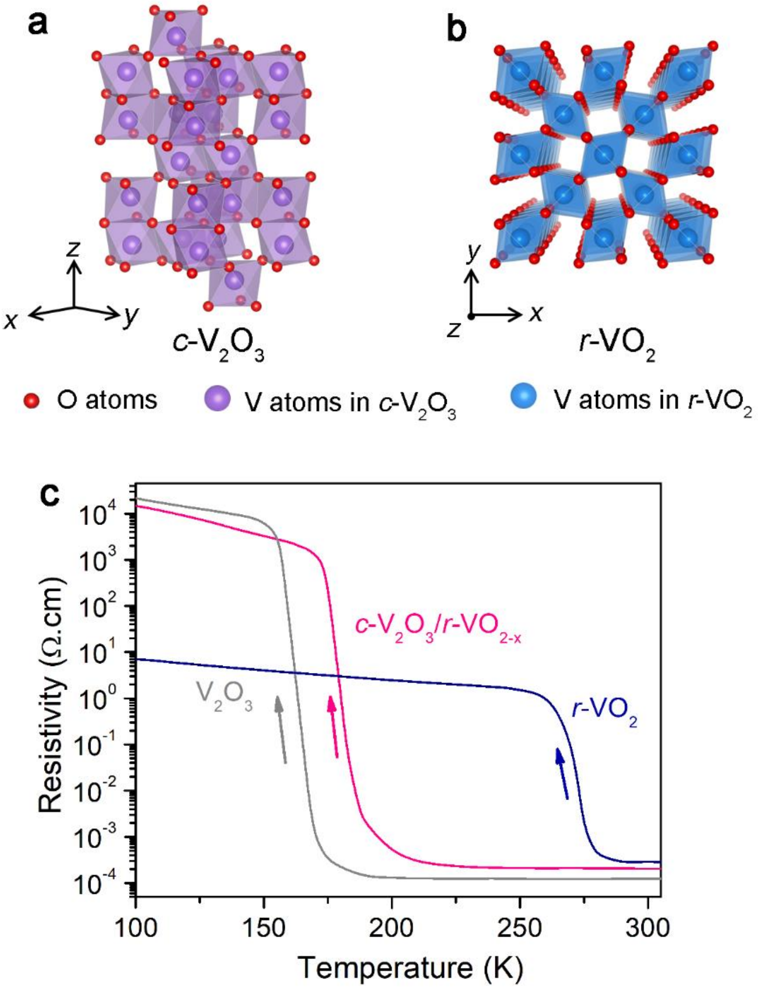


**Supplementary Figure 1. Crystallographic structures of *c*-$V_2O_3$, *r*-$VO_2$, and their electronic properties. a**, **b**, Atomic structures of corundum $V_2O_3$ (a) and rutile $VO_2$ (b). **c**, Temperature dependence of the resistivity of *c*-$V_2O_3$, *c*-$V_2O_3$/*r*-$VO_{2-x}$ ($x = 0.22$) and *r*-$VO_2$ films grown on $Al_2O_3$ single-crystalline substrates.

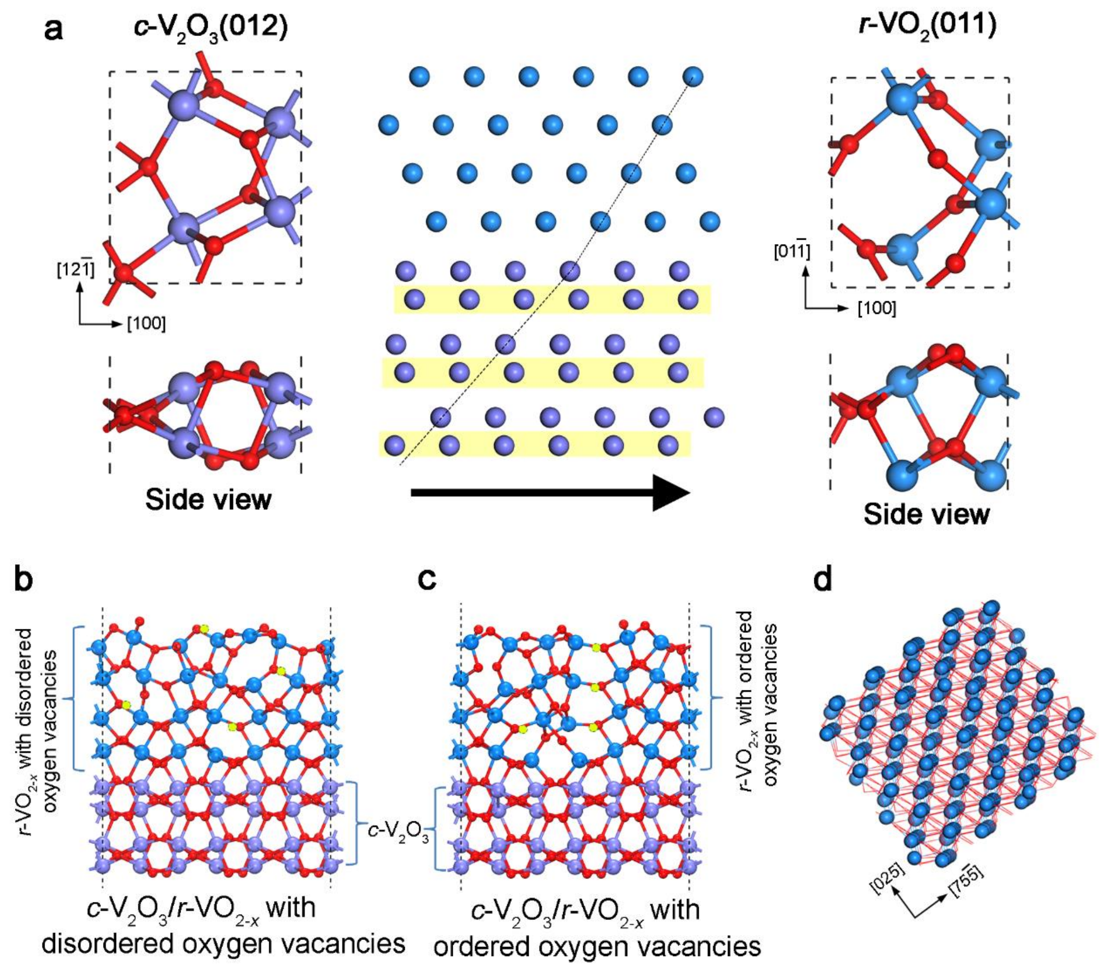


**Supplementary Figure 2. DFT calculation for corundum-to-rutile phase transformation. a**, Atomic model for the corundum-to-rutile phase transformation from $c$-$V_2O_3$ to $r$-$VO_{2-x}$. **b**, **c**, Relaxed atomic structures of $c$-$V_2O_3$/$r$-$VO_{2-x}$ with disordered (b) and ordered oxygen vacancies (a). Purple and blue balls represent vanadium atoms in corundum-type $V_2O_3$ and rutile-type $r$-$VO_{2-x}$, red and yellow ones denote oxygen atoms and ordered oxygen vacancies. The adhesion work $W_{ad}$, which is defined as the reversible work needed to separate an interface into two free surface, is employed to evaluate the thermodynamic stability of $c$-$V_2O_3$/$r$-$VO_{2-x}$ systems with disordered or ordered oxygen vacancies. Here the value of $W_{ad\text{-}order}$ for $c$-$V_2O_3$/$r$-$VO_{2-x}$ with ordered oxygen vacancies is determined to be 1.614 J m$^{-2}$ according to the equation: $W_{ad} = (E_{c\text{-}V2O3}+E_{r\text{-}VO2\text{-}x}-E_{c\text{-}V2O3/r\text{-}VO2\text{-}x})/A_i$. Here $E_{c\text{-}V2O3}$ and $E_{r\text{-}VO2\text{-}x}$ are the total energy of a relaxed, isolated $c$-$V_2O_3$ and $r$-$VO_{2-x}$ slabs with disordered or ordered oxygen defects, $E_{c\text{-}V2O3/r\text{-}VO2\text{-}x}$ is the total energy of $c$-$V_2O_3$/$r$-$VO_{2-x}$ system with disordered or ordered oxygen defects, $A_i$ is the interface area. This value is higher than the ones for

$c$-$V_2O_3$/$r$-$VO_{2-x}$ with disordered oxygen vacancies ($W_{ad\text{-}disorder}$ = 1.512 J $m^{-2}$) and $c$-$V_2O_3$/$r$-$VO_2$ without oxygen defects ($W_{ad}$ = 1.471 J $m^{-2}$), which indicates that it is more thermodynamically favorable for the ordered oxygen vacancies to form in the $c$-$V_2O_3$/$r$-$VO_{2-x}$. **d**, Rotated atomic structures of $r$-$VO_{2-x}$ layer with ordered oxygen defects (Figure 1b), which corresponds to HRTEM image of $r$-$VO_{2-x}$ layer shown in Figure 1g.

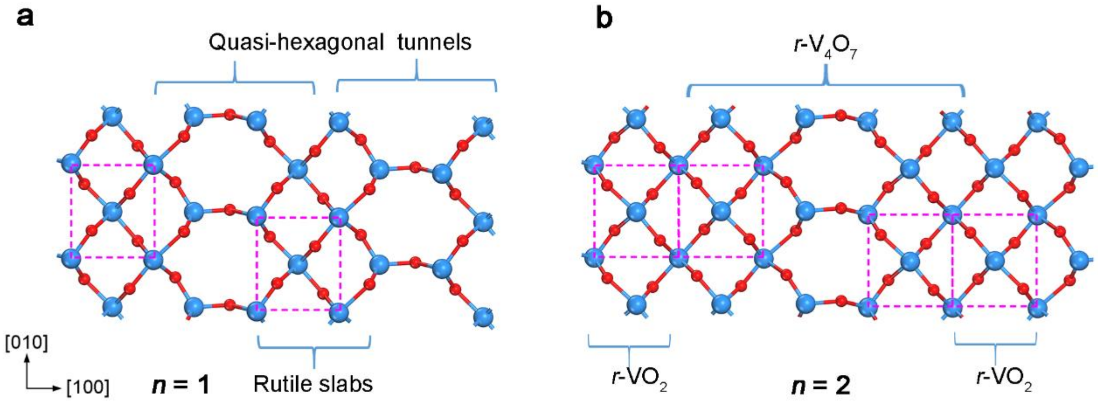


**Supplementary Figure 3. Representative *r*-$VO_{2\text{-}x}$ atomic structure. a, b,** Atomic schematics for the ideal structures of *r*-$VO_{2\text{-}x}$ with $x$ = 0.25 (a) and 0.167 (b), which consist of quasi-hexagonal tunnels and rutile slabs.

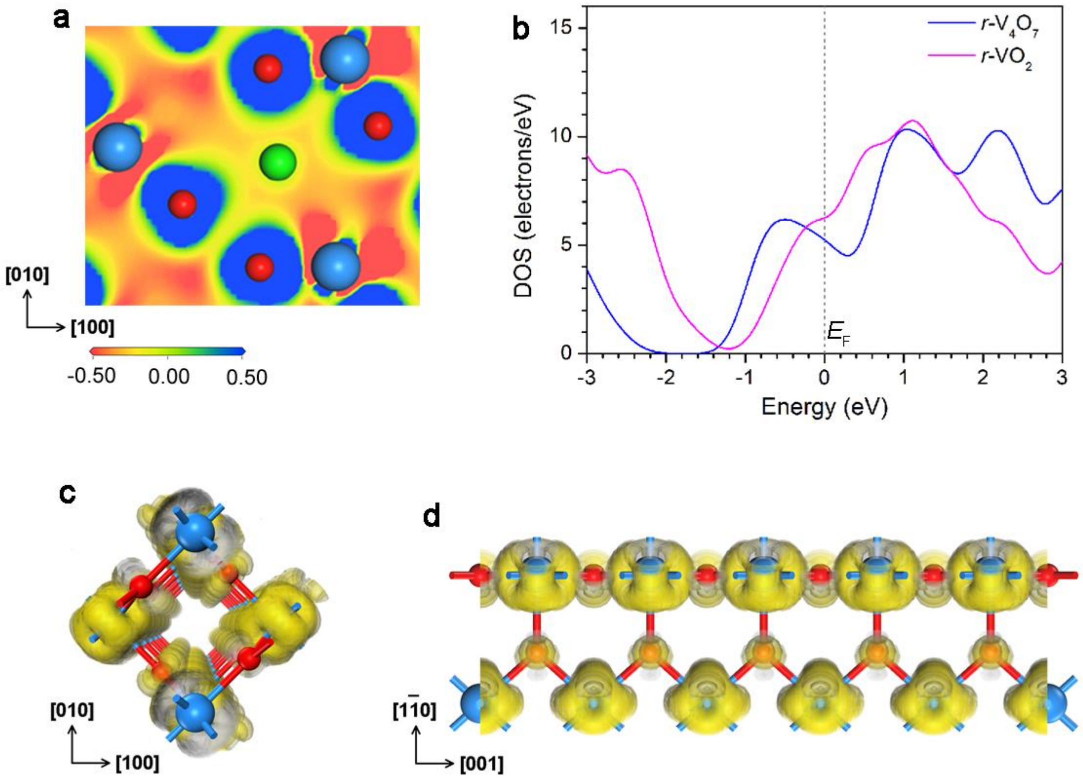


**Supplementary Figure 4. Ion and electron transport properties of *r*-$VO_{2-x}$ and *r*-$VO_2$.** **a**, Possible sodium intercalation sites in the quasi-hexagonal tunnels of the *r*-$VO_{2-x}$ with an electron density difference with respect to the sum of atomic densities. Green ball denotes the Na cation. **b**, Density of states for *r*-$VO_2$ and *r*-$V_4O_7$ nearby Fermi level. **c**,**d**, Electron density distribution of the projected orbitals for the rutile slabs of the pristine *r*-$VO_2$ within the energy range $E_F$ - 0.8 eV < $E$ < $E_F$ + 0.8 eV. The isosurface value is 0.01 e/Å$^3$.

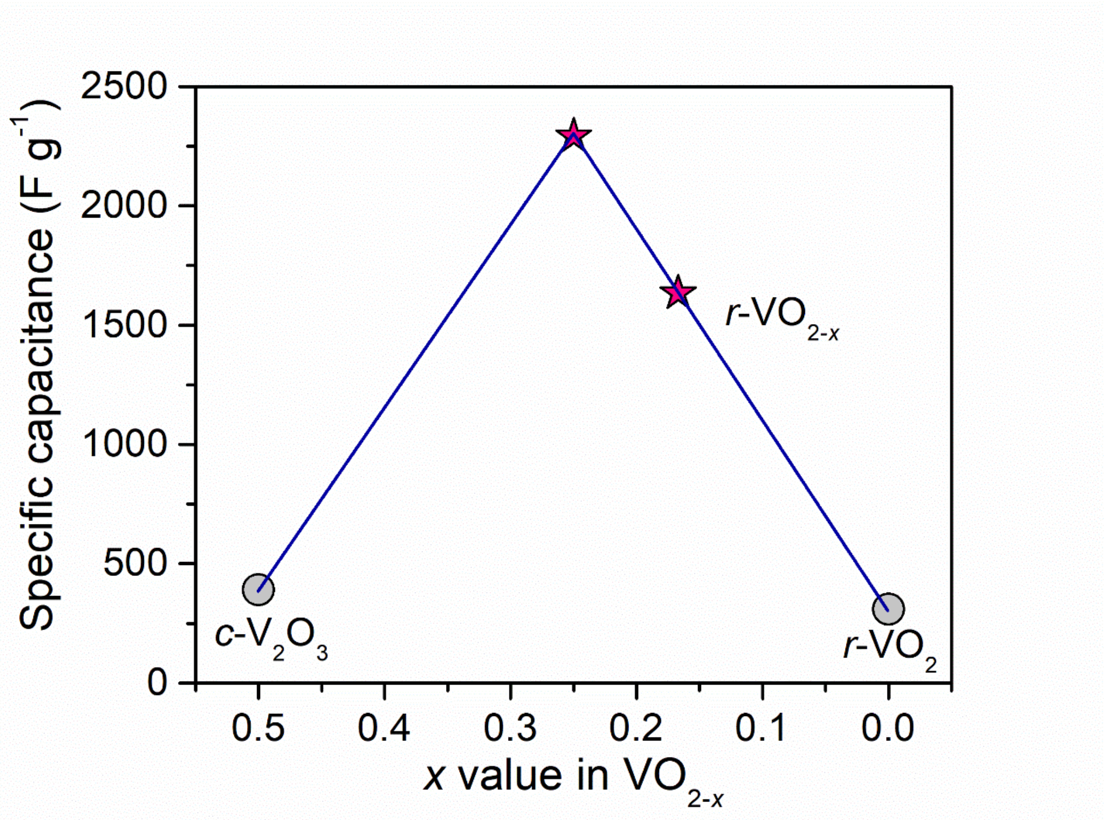


**Supplementary Figure 5. Phase diagram for specific capacitance of vanadium oxides as a function of *x* value in $VO_{2-x}$.** Here the *r*-$V_4O_7$ is predicted to have the highest specific capacitance (1528.6 F $g^{-1}$) because of all V cations that can react with Na ions in a voltage window of 0.8 V. While the *r*-$V_6O_{11}$ only has its two-third V atoms to take part in the redox reaction with Na ions, its theoretical specific capacitance is 1002.1 F $g^{-1}$. Considering that the redox reaction only occurs on the surface of *c*-$V_2O_3$ and *r*-$VO_2$, their specific capacitance depends on their specific surface area and are obtained by experimental measurement of NP *c*-$V_2O_3$ and *r*-$VO_2$ electrodes. The solid lines denotes the specific capacitance for the mixtures of *c*-$V_2O_3$ and *r*-$V_4O_7$, *r*-$VO_2$ and *r*-$V_4O_7$ because of the CTR phase transformation.

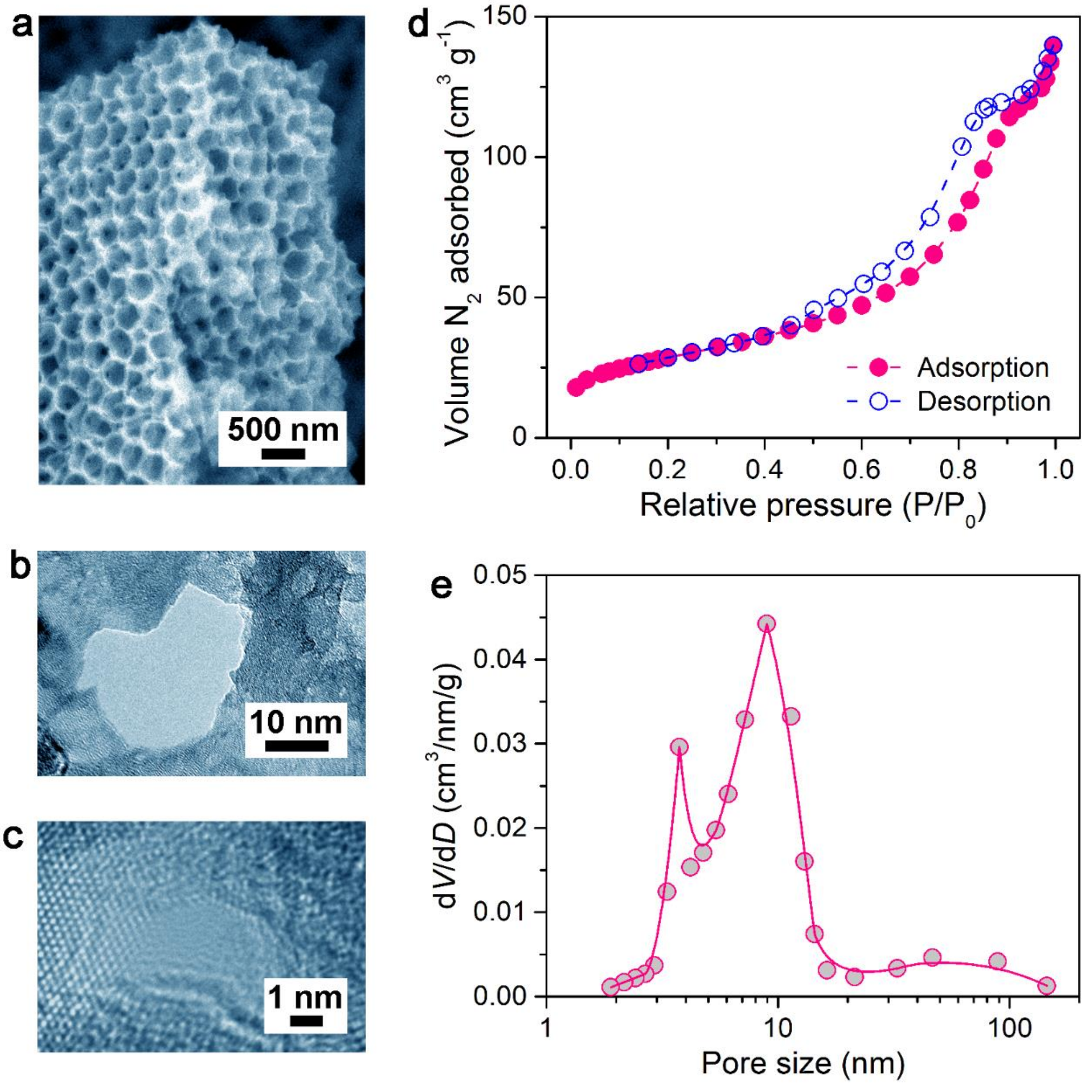


**Supplementary Figure 6**. **Morphology of multimodal nanoporous vanadium oxides.** **a**, Cross-section SEM image of 3D NP *c*-$V_2O_3$/*r*-$VO_{2-x}$ films fabricated by in-situ thermal oxidation of the precursor *c*-$V_2O_3$ scaffolds with thickness of ~1.2 μm for 10 min. **b**, **c**, Bright-field TEM images of NP *c*-$V_2O_3$/*r*-$VO_{2-x}$ films showing the hierarchical porous structure consisting of ~20 nm mesopores (b), ~2 nm micropores (c) in walls. **d**, $N_2$ adsorption-desorption isotherms for hierarchical NP *c*-$V_2O_3$/*r*-$VO_{2-x}$ with BET specific area of 99.8 $m^2$ $g^{-1}$. **e**, Multimodal pore size distributions of NP *c*-$V_2O_3$/*r*-$VO_{2-x}$ films.

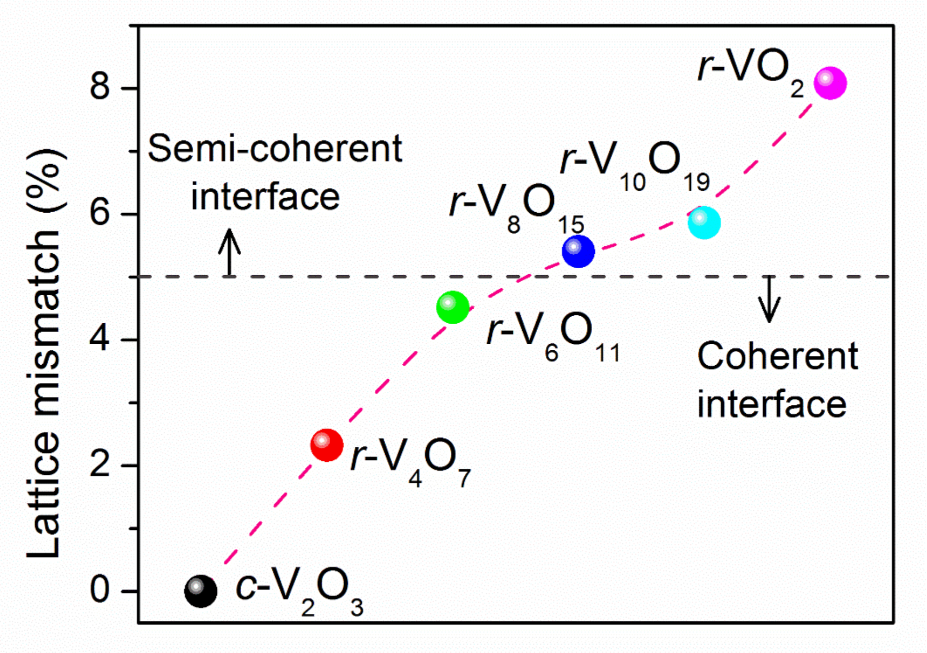


**Supplementary Figure 7. Lattice mismatches of metastable *r*-$VO_{2-x}$** ($x = 0.25, 0.167, 0.125, 0.1$) and *r*-$VO_2$ ($n \to \infty$) with respect to *c*-$V_2O_3$ substrate.

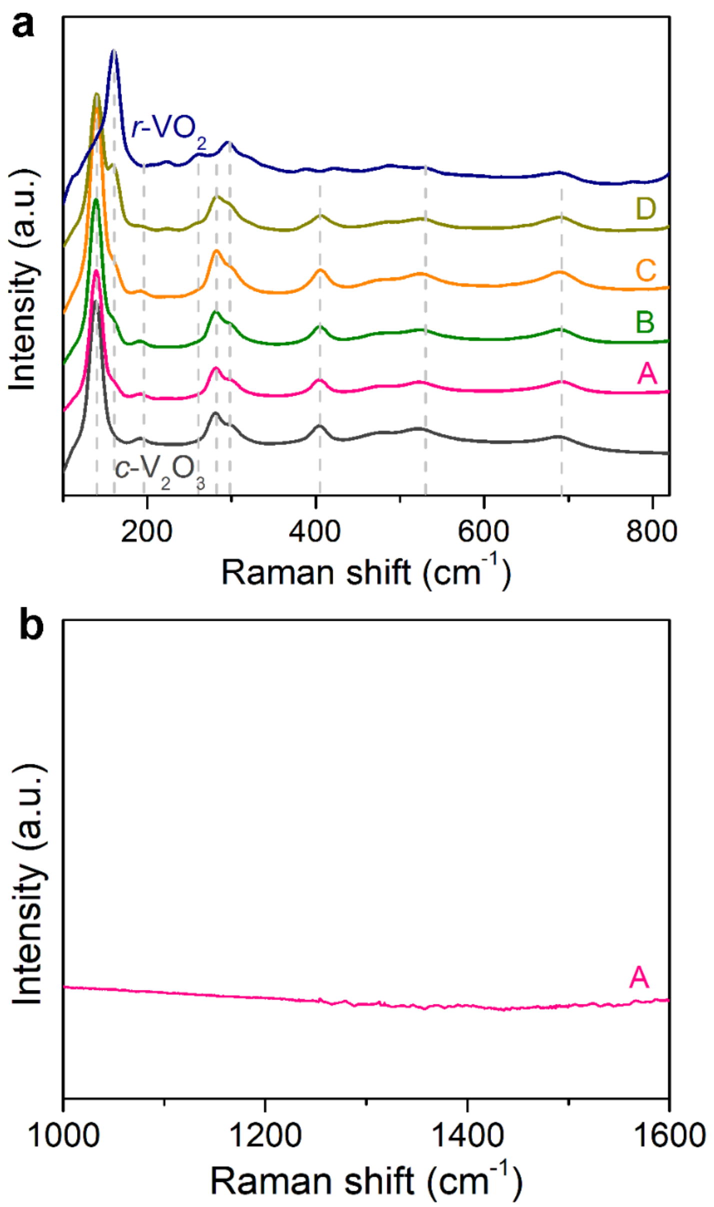


**Supplementary Figure 8. Raman characterization on vanadium oxide films at different thermal oxidation time. a,** Raman spectra for NP $c$-$V_2O_3$, $r$-$VO_2$, and $c$-$V_2O_3$/$r$-$VO_{2-x}$ films, demonstrating the evolution of phase transformation via thermal oxidation of the corundum $c$-$V_2O_3$ at 300 °C for 10 (A), 20 (B), 30 (C) and 60 min (D), respectively. **b**, Raman spectrum of NP $c$-$V_2O_3$/$r$-$VO_{2-x}$ film with the thermal oxidation of 10 min. There are not characteristic Raman peaks to be observed in the range from 1300 to 1600 $cm^{-1}$, demonstrating that the selective removal of PS nanospheres by calcination does not give rise to the formation of carbon.

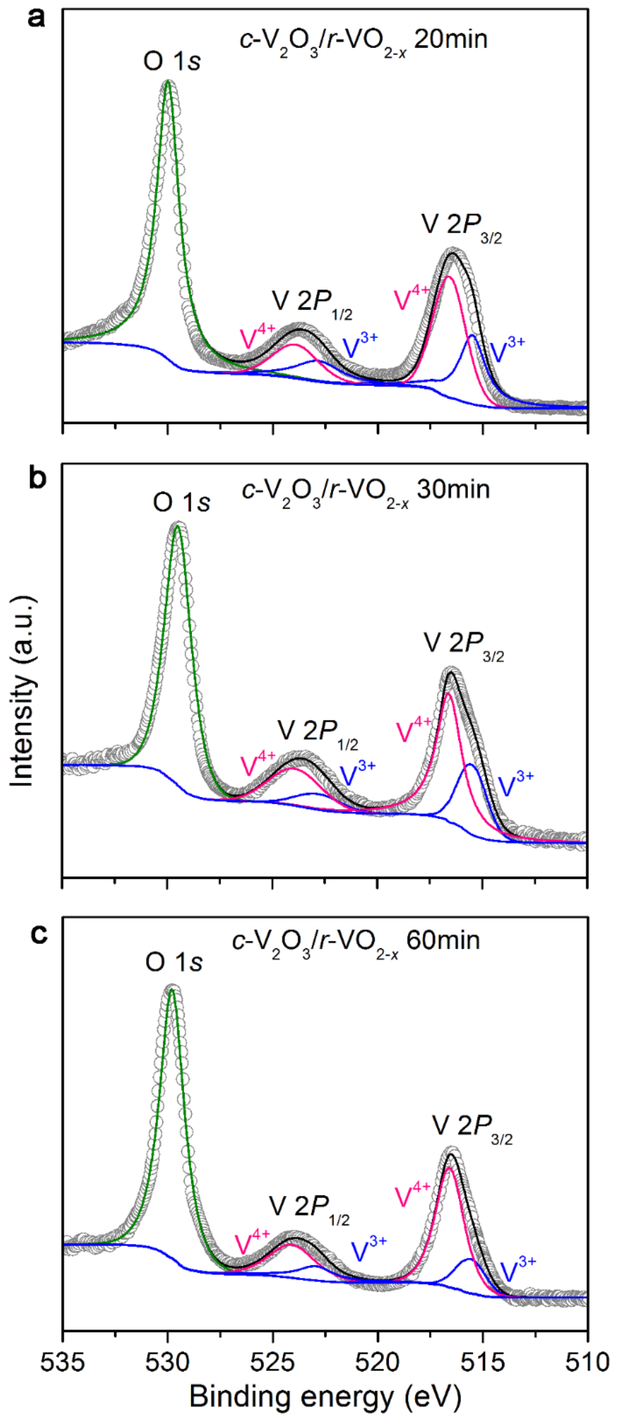


**Supplementary Figure 9**. **High-resolution XPS spectra for NP *c*-$V_2O_3$/*r*-$VO_{2-x}$ films**. NP *c*-$V_2O_3$/*r*-$VO_{2-x}$ films are fabricated by thermal oxidation of the precursor *c*-$V_2O_3$ scaffolds at 300 °C for, **a**, 20, **b**, 30 and, **c**, 60 min in a tube furnace sealed with ambient air.

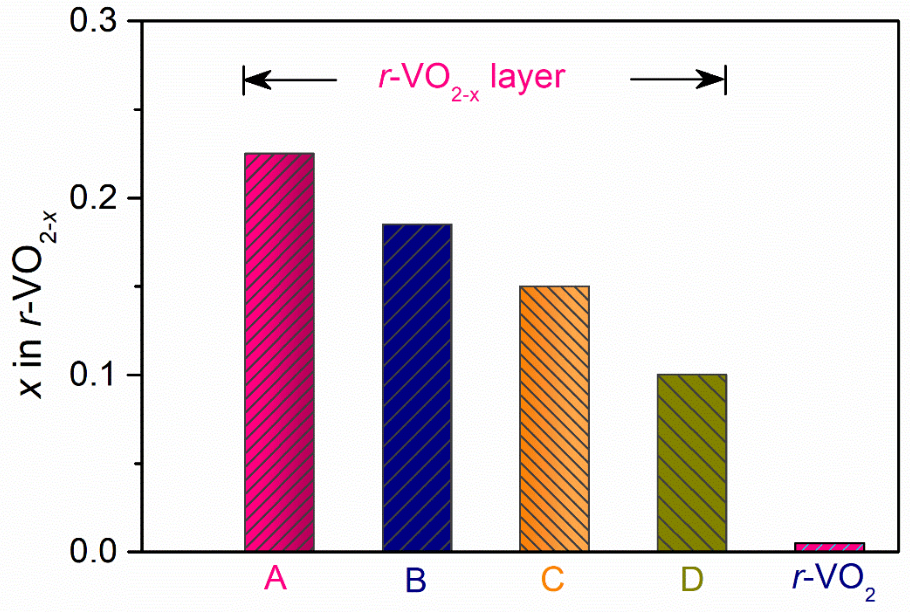


**Supplementary Figure 10. The *x* values of rutile-type crystalline *r*-$VO_{2\text{-}x}$ layers.** The *r*-$VO_{2\text{-}x}$ layers that form via phase transformation from the *c*-$V_2O_3$ at different thermal oxidation time: 10 (A), 20 (B), 30 (C) and 60 min (D).

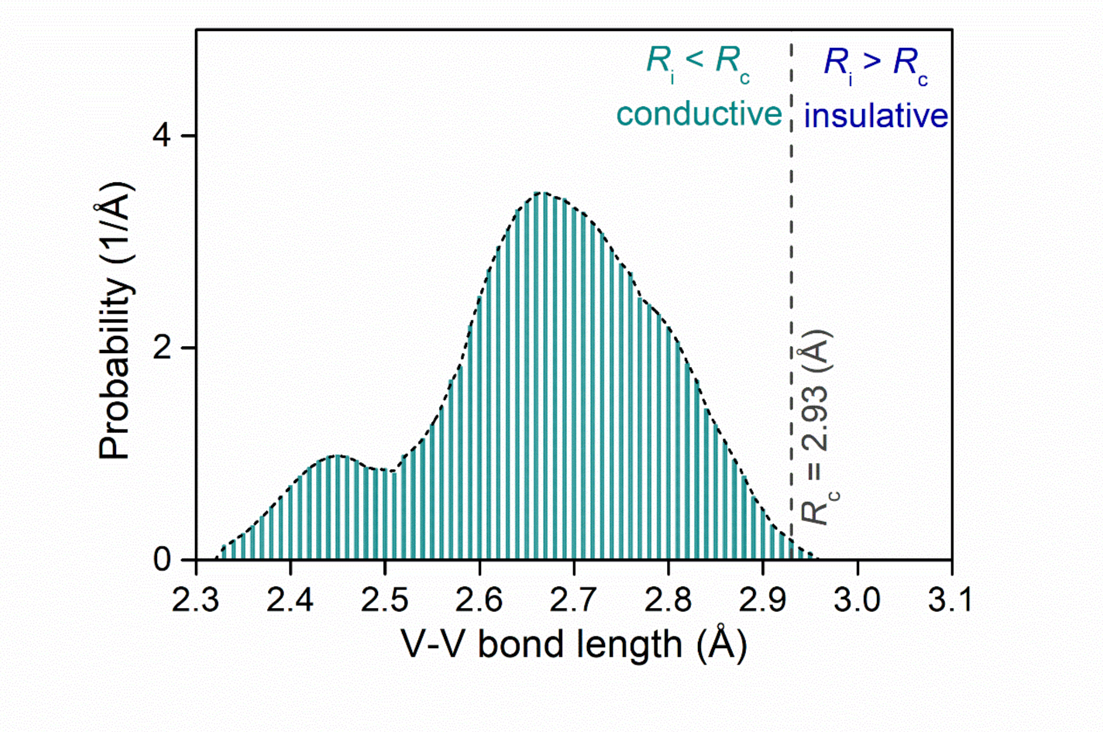


**Supplementary Figure 11. V-V bond lengths.** A distribution of V-V bond lengths at the *c*-$V_2O_3$(012)/*r*-$VO_{2-x}$(011) interface range ($R_i$). The vertical dashed line denotes the critical V-V bond length, $R_c$. When $R_i < R_c$, the V-V chains are conductive; while $R_i > R_c$, the ones are insulative.

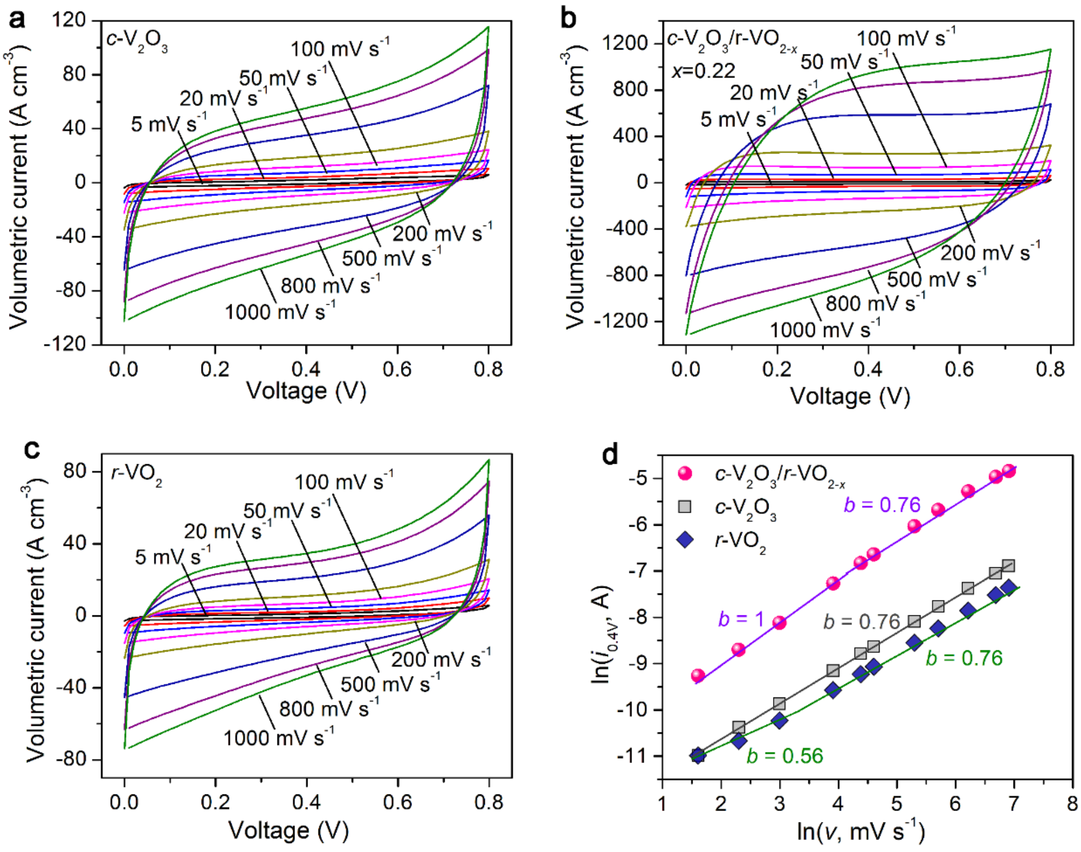


**Supplementary Figure 12**. **Electrochemical properties of NP *c*-$V_2O_3$, NP *c*-$V_2O_3$/*r*-$VO_{2-x}$ and NP *r*-$VO_2$**. CV curves in the potential window of 0–0.8 V at various scan rates for, **a**, NP *c*-$V_2O_3$, **b**, NP *c*-$V_2O_3$/*r*-$VO_{2-x}$ ($x$ = 0.22) and, **c**, NP *r*-$VO_2$ film electrodes. **d**, Logarithmic plot of the scan rate versus the discharge current at the voltage of 0.2 V for NP *c*-$V_2O_3$, *c*-$V_2O_3$/*r*-$VO_{2-x}$ ($x = 0.22$) and *r*-$VO_2$ films to determine the $b$ values assuming the power-law relationship between the current and the scan rate, $i = av^b$.

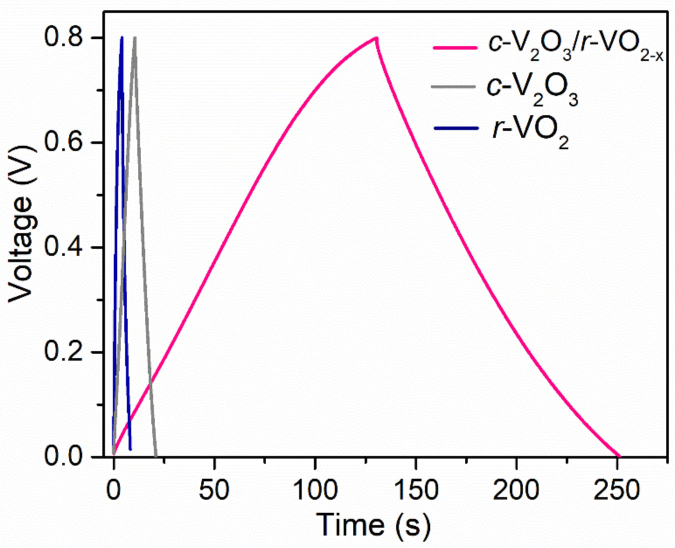


**Supplementary Figure 13**. **Voltage-time profiles for NP *c*-$V_2O_3$, NP *c*-$V_2O_3$/*r*-$VO_{2-x}$ and NP *r*-$VO_2$** Galvanostatic charge/discharge curves for NP *c*-$V_2O_3$, NP *c*-$V_2O_3$/*r*-$VO_{2-x}$ ($x = 0.22$) and NP *r*-$VO_2$ film electrodes at the current density of 10.4 A $cm^{-3}$.

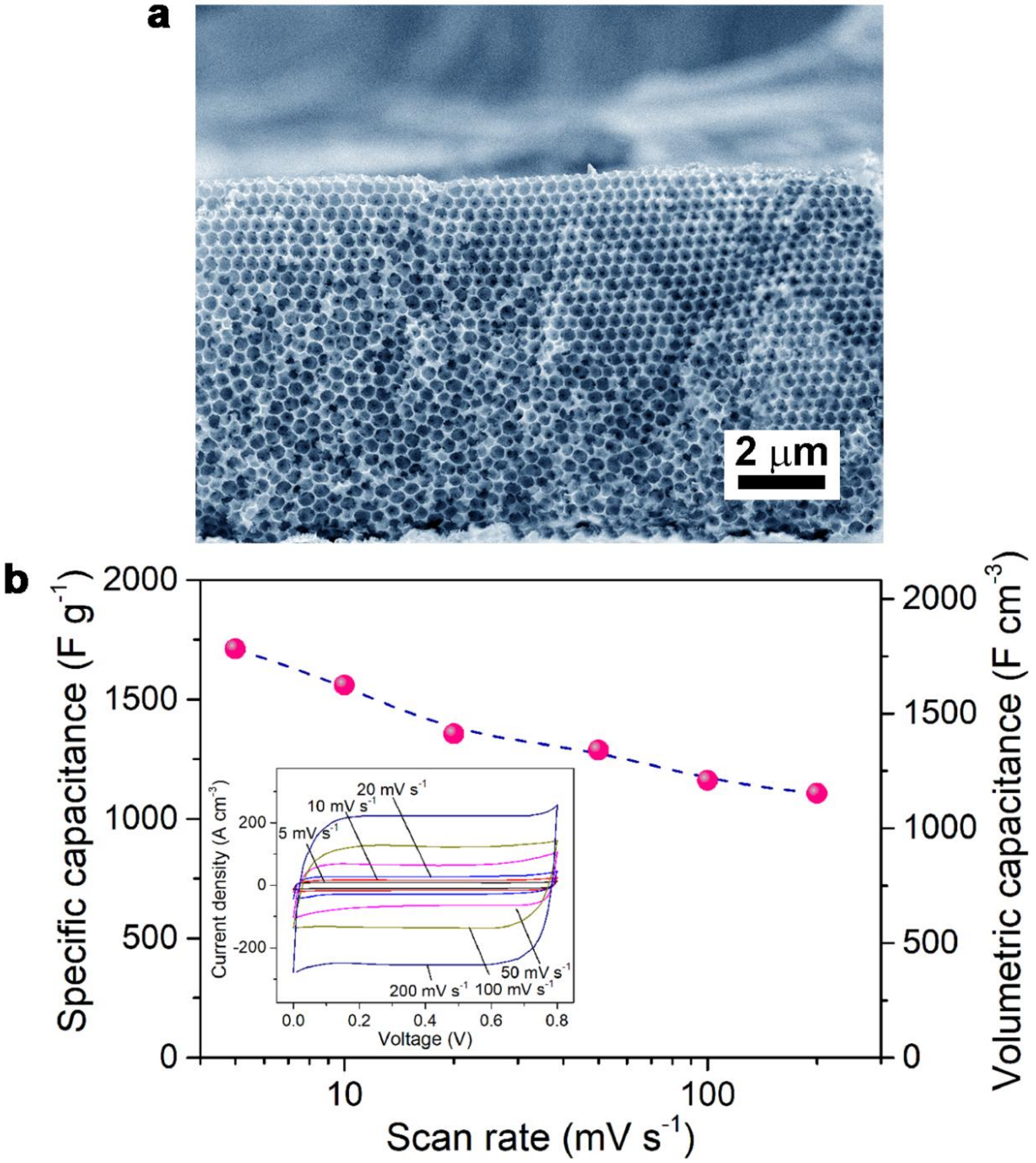


**Supplementary Figure 14. Structural and electrochemical properties of thick NP *c*-$V_2O_3$/*r*-$VO_{2-x}$ films.** **a**, Typical cross-section SEM image of NP *c*-$V_2O_3$/*r*-$VO_{2-x}$ (thermal oxidation time of 10 min) with thickness of ~7.8 μm. **b**, Specific and volumetric capacitances of 7.8-μm-thick NP *c*-$V_2O_3$/*r*-$VO_{2-x}$ film electrode with a thermal oxidation time of 10 min at various scan rates. Inset: Typical CV curves of 7.8-μm-thick NP *c*-$V_2O_3$/*r*-$VO_{2-x}$ film electrode at various scan rates, which are collected in three-electrode setup in a voltage window of 0-0.8 V.

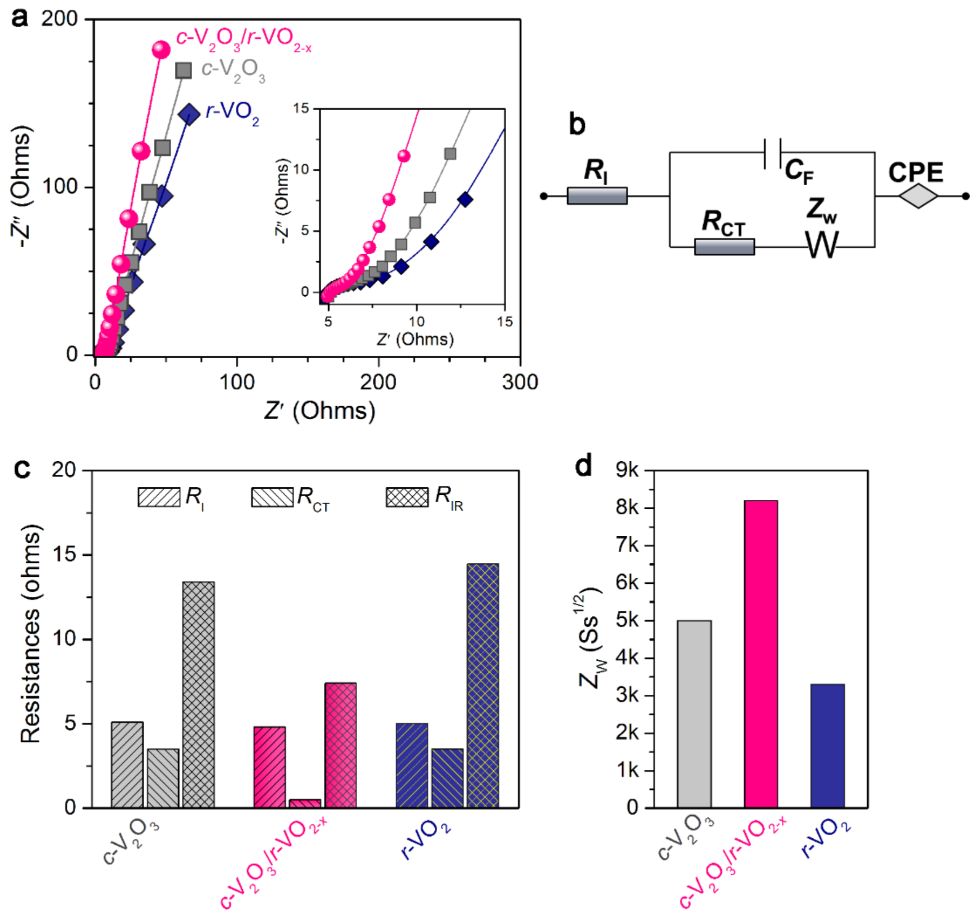


**Supplementary Figure 15**. **EIS analysis of NP *c*-$V_2O_3$, *c*-$V_2O_3$/*r*-$VO_{2\text{-}x}$ and NP *r*-$VO_2$ electrodes. a**, Comparison of EIS spectra for NP *c*-$V_2O_3$, *r*-$VO_2$ and *c*-$V_2O_3$/*r*-$VO_{2\text{-}x}$ electrodes. Inset: A magnification of EIS in the high- to middle-frequency range. **b**, The electrical equivalent circuit used for fitting EIS spectra. **c**, Comparison of $R_I$, $R_{CT}$ and $R_{IR}$ values for NP *c*-$V_2O_3$/*r*-$VO_{2\text{-}x}$, *c*-$V_2O_3$ and *r*-$VO_2$ electrodes. **d**, Comparison of $Z_w$ values for NP *c*-$V_2O_3$, *r*-$VO_2$ and *c*-$V_2O_3$/*r*-$VO_{2\text{-}x}$ electrodes.

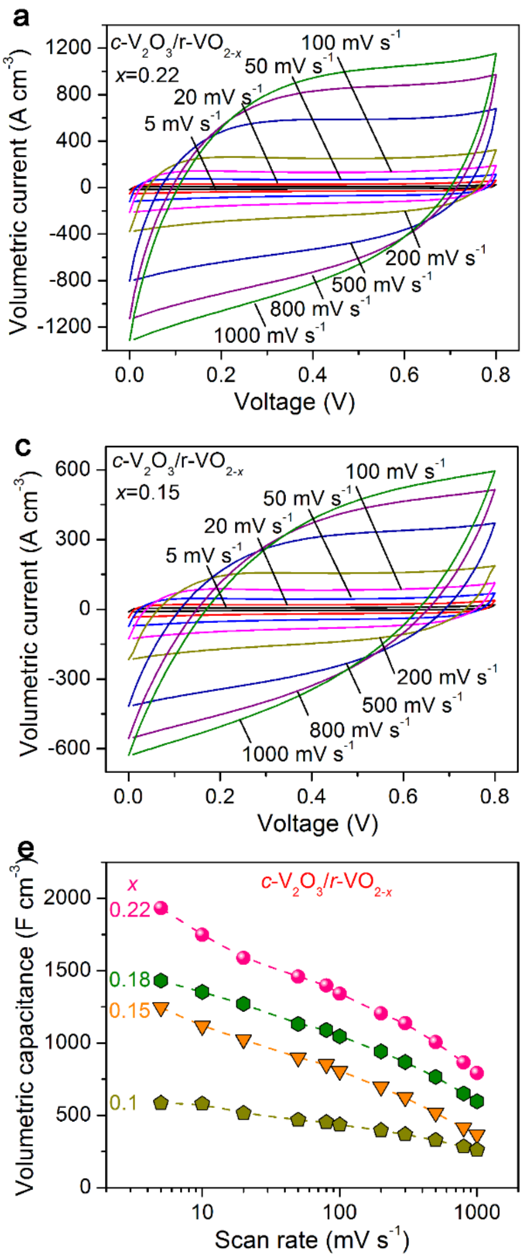


**Supplementary Figure 16. Electrochemical properties of NP *c*-$V_2O_3$/*r*-$VO_{2-x}$ electrodes.** CV curves in the potential window of 0 - 0.8 V at various scan rates for NP *c*-$V_2O_3$/*r*-$VO_{2-x}$ film electrodes with, **a**, $x = 0.22$, **b**, $x = 0.18$, **c**, $x = 0.15$ and, **d**, $x = 0.10$, respectively. **e**, Volumetric capacitances at various scan rates for NP *c*-$V_2O_3$/*r*-$VO_{2-x}$ film electrodes with $x = 0.22$, 0.18, 0.15 and 0.10.

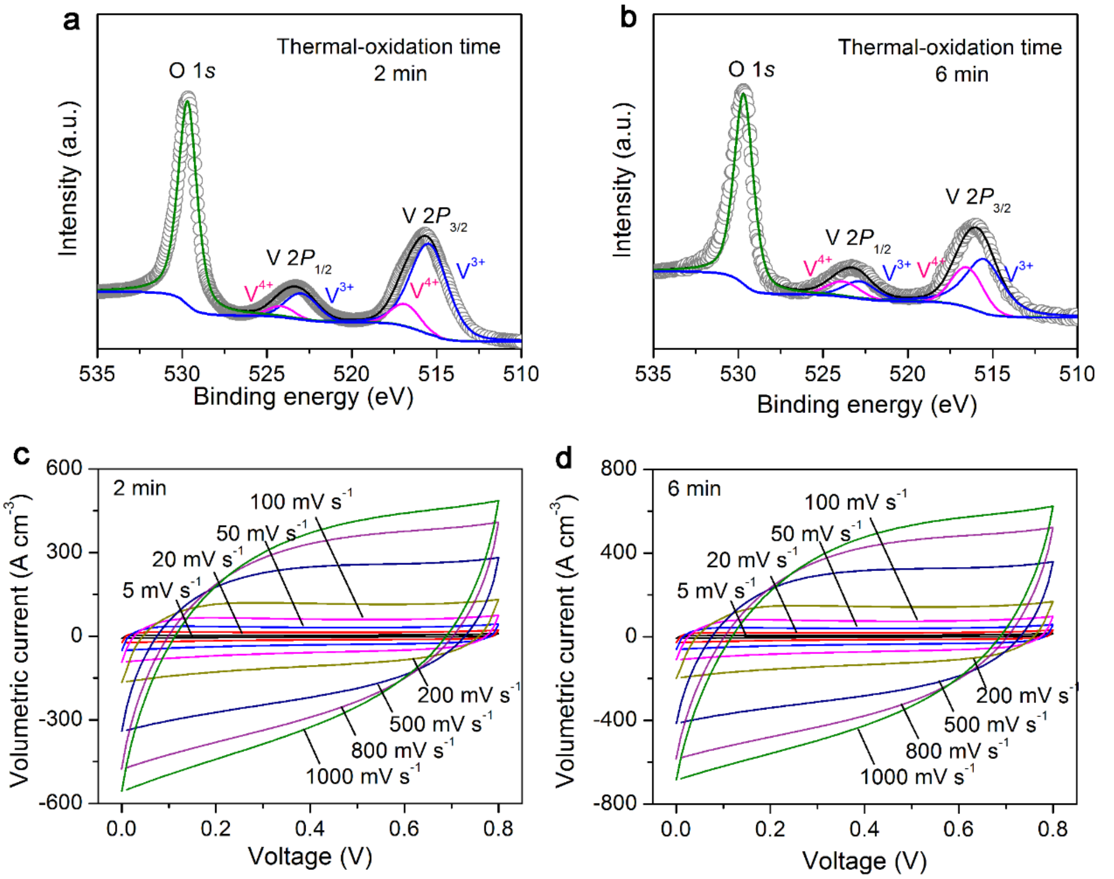


**Supplementary Figure 17. XPS measurements and electrochemical characterization for NP *c*-$V_2O_3$ with short thermal oxidation time. a,b,** High-resolution XPS spectra for NP *c*-$V_2O_3$ films after thermal oxidation for 2 (a) and 6 min (b) in a tube furnace sealed with ambient air. Here the $x$ values are determined to be 0.428 and 0.337 according to the ratio of $V^{3+}/V^{4+}$ at the V $2p_{3/2}$ peaks for thermal-oxide time of 2 and 6 min, respectively. **c, d**, CV curves in the potential window of 0 - 0.8 V at various scan rates for NP *c*-$V_2O_3$/*r*-$VO_{2-x}$ film electrodes with $x = 0.428$ (c) and 0.337 (d).

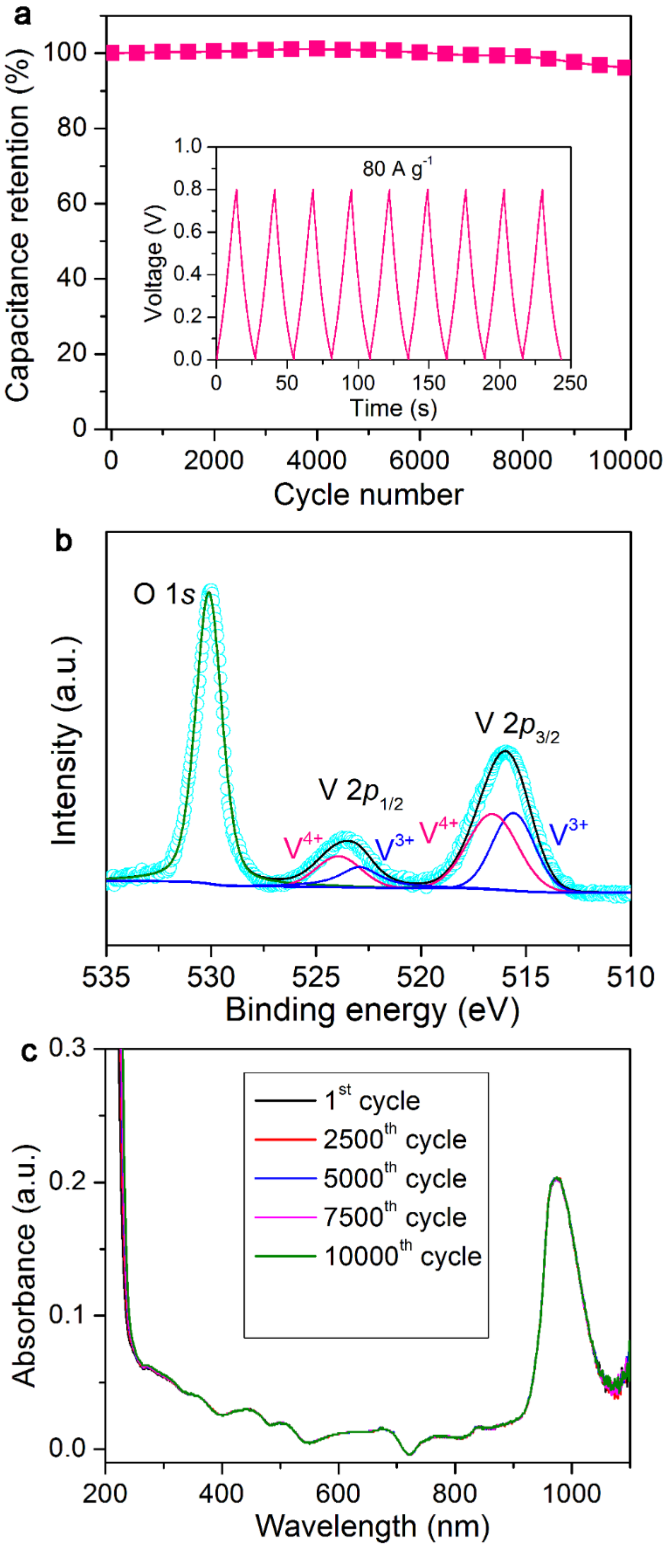


**Supplementary Figure 18. Stability measurements of NP *c*-$V_2O_3$/*r*-$VO_{2-x}$ electrode.** **a**, Electrochemical durability of NP *c*-$V_2O_3$/*r*-$VO_{2-x}$ with $x$ = 0.22 in a long-term galvanostatic charge/discharge cycles. Inset: Typical galvanostatic charge/discharge curves at a current density of 80 A $g^{-1}$. **b**, XPS spectrum of NP *c*-$V_2O_3$/*r*-$VO_{2-x}$ with $x$ =

0.22 after 10000 cycles of galvanostatic charge/discharg. **c**, UV-Visible spectra of $Na_2SO_4$ electrolytes, in which the stability measurement of NP *c*-$V_2O_3$/*r*-$VO_{2-x}$ electrode is performed via the galvanostatic charge/discharge, at 1$^{st}$, 2500$^{th}$, 5000$^{th}$, 7500$^{th}$ and 10000$^{th}$ cycles.

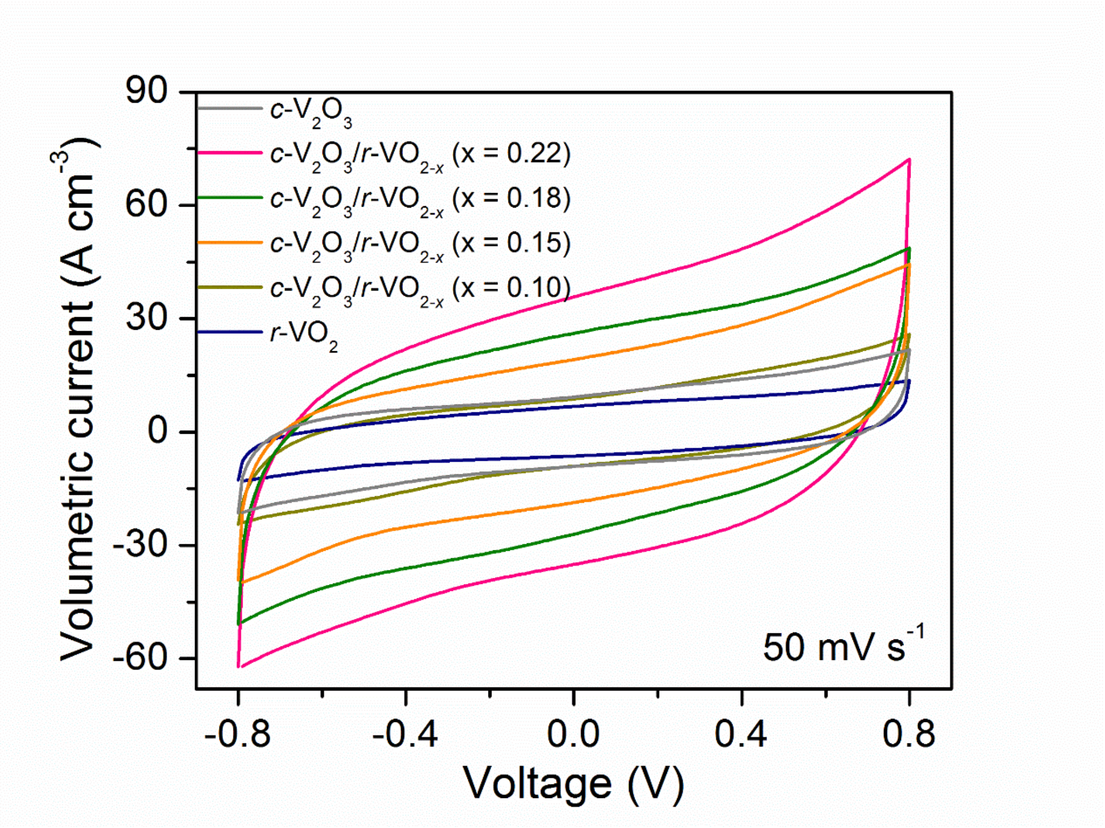


**Supplementary Figure 19. Bipolar electrochemical properties of nanoporous vanadium oxide electrodes.** CV curves in the potential window of -0.8 - 0.8 V at various scan rates for NP *c*-$V_2O_3$, NP *r*-$VO_2$ and NP *c*-$V_2O_3$/*r*-$VO_{2-x}$ film electrodes with $x$ = 0.22, 0.18, 0.15 and 0.10, respectively.

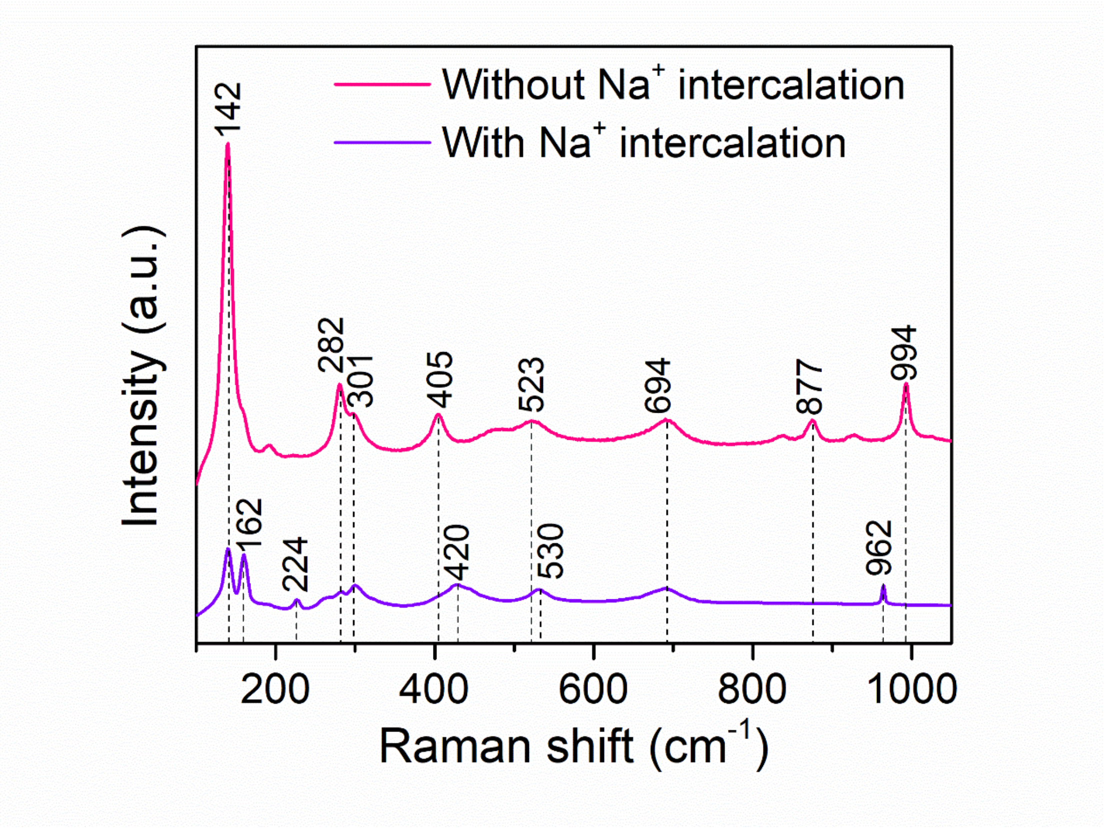


**Supplementary Figure 20. Comparison of Raman spectra for vanadium oxide with and without $Na^+$ intercalation.** Raman spectra for NP *c*-$V_2O_3$/*r*-$VO_{2-x}$ films (thermal oxidation time of 10 min) before and after $Na^+$ intercalation. Compared with the Raman spectrum of NP *c*-$V_2O_3$/*r*-$VO_{2-x}$ films without $Na^+$ intercalation, there is a new characteristic Raman peak at 962 $cm^{-1}$ for the ones with $Na^+$ intercalation. The presence of such line in the frequency region related to vanadyl stretching modes suggests strong interaction of sodium with the apical oxygen atoms in the *r*-$VO_{2-x}$ tunnels. In addition, the $Na^+$ intercalation results in new peak at 224 $cm^{-1}$ and appearance of shoulders at 420 and 162 $cm^{-1}$.

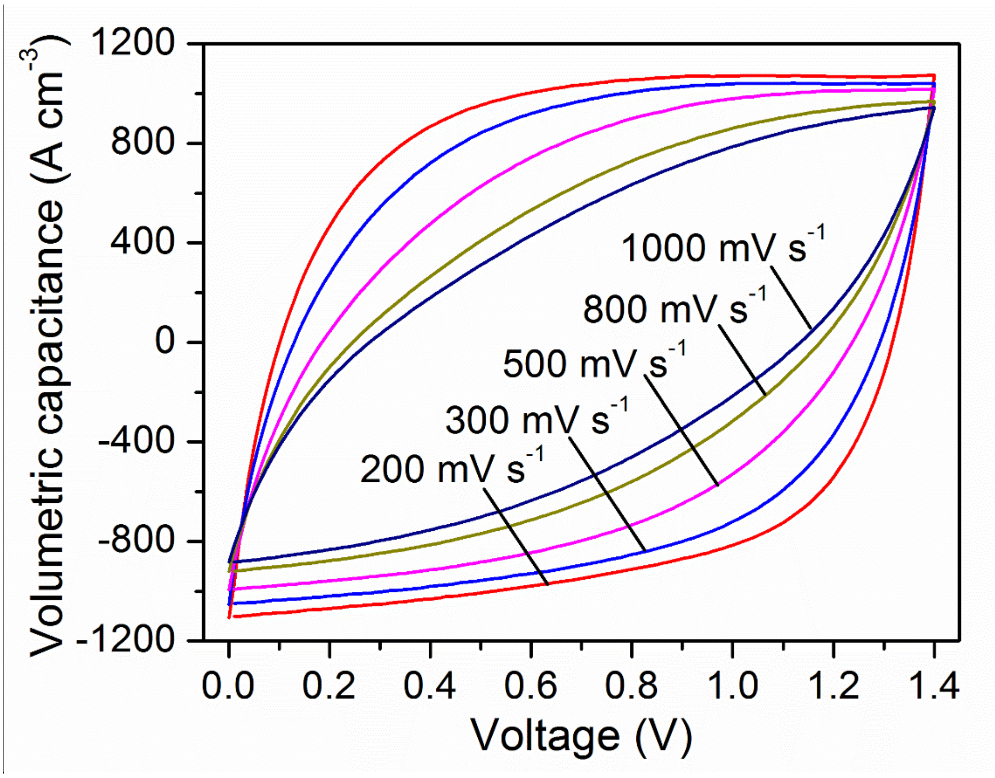


**Supplementary Figure 21. Electrochemical characterization of devices at high scan rates.** CV curves for symmetric pseudocapacitors based on NP *c*-$V_2O_3$/*r*-$VO_{2-x}$ ($x$ = 0.22) electrodes at scan rates from 200 to 1000 mV $s^{-1}$ in the potential window from 0 to 1.4 V.

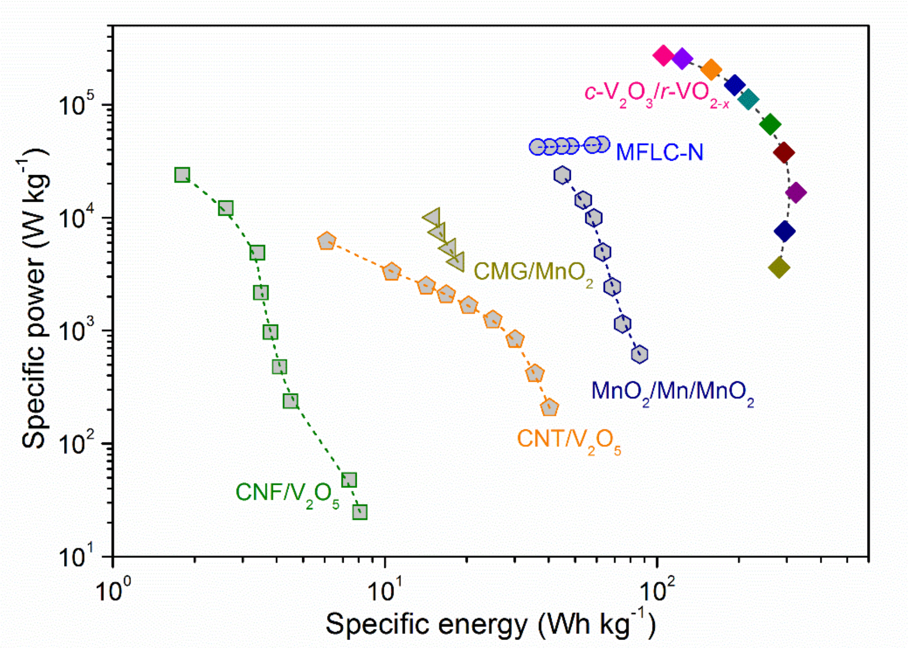


**Supplementary Figure 22. Comparison of energy and power densities.** Ragone plot of specific energy versus specific power for NP $c$-$V_2O_3$/$r$-$VO_{2-x}$ electrodes in a potential window of 1.4 V, as well as materials based on MFLC-N[1], $MnO_2$/Mn/$MnO_2$ nanotube arrays[2], chemically modified graphene (CMG)/$MnO_2$[3], CNT/$V_2O_5$ [4], and carbon nanofiber (CNF)/$V_2O_5$ [5].

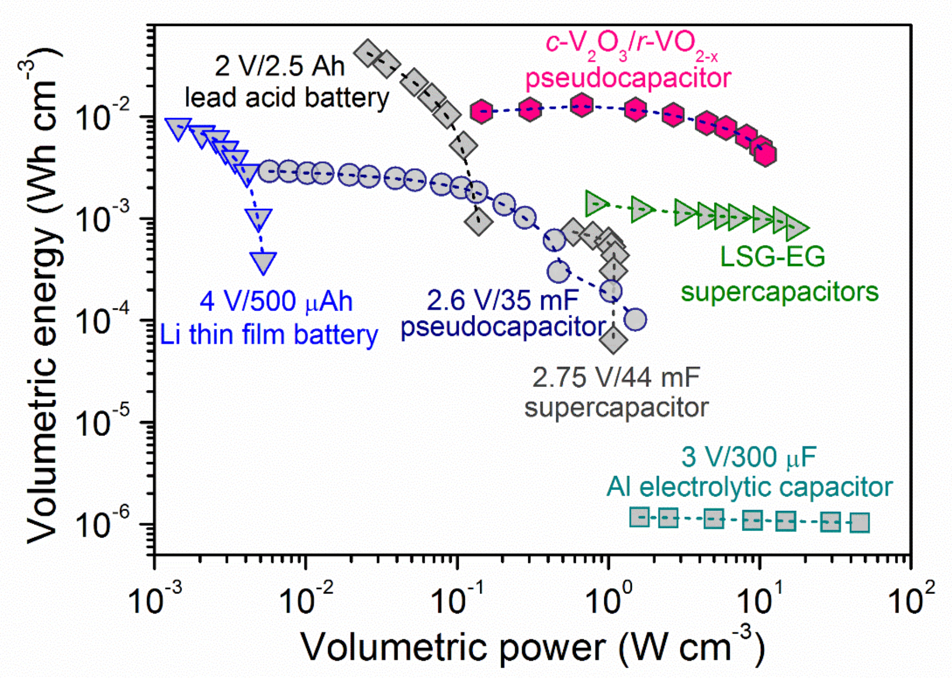


**Supplementary Figure 23**. **Volumetric energy and power densities for devices.** Volumetric power and energy densities in Ragone plot for symmetric NP *c*-$V_2O_3$/*r*-$VO_{2-x}$-based devices (which is constructed with a cotton paper with thickness of 40 μm as a separator and two SS sheets with thickness of 10 μm as current collectors), comparing with commercially available devices: 4 V/500 μAh Li thin film battery[6], 2 V/2.5 Ah lead acid battery[7], 2.75 V/44 mF activated carbon supercapacitor[8], 2.6 V/35 mF pseudocapacitors[7], 3 V/300 μF Al electrolytic capacitor[7], in addition to a laser-scribed graphene supercapacitor (LSG-EG) in ionic liquid previously reported in literature[9].

## Supplementary Notes

**Supplementary Note 1.** Electrochemical calculations. The specific capacitance ($C_s$, in F g$^{-1}$) and volumetric capacitance ($C_V$, in F cm$^{-3}$) were calculated by integrating the CVs in terms of Supplementary Equation (1)

$$C_s = Q/(\Delta E m),\ C_V = Q/(\Delta E V) \quad (1)$$

where $Q = (\int_{E_1}^{E_2} i(E)dE/v + \int_{E_1}^{E_2} i(E)dE/v)/2$ in the potential window of -0.8—0.8 V with $E_2$ = 0.8 V and $E_1$ = 0 V, and $Q = \int_{E_1}^{E_2} i(E)dE/v$ in the potential range from 0 to 1.4 V with $E_2$ = 1.4 V and $E_1$ = 0 V. Here $m$ and $V$ are the mass and geometric volume of single electrode for NP $c$-$V_2O_3$, $c$-$V_2O_3$/$r$-$VO_{2-x}$ and $r$-$VO_2$ films, $i(E)$ is the current as a function of potential $E$ at the scan rate ($v$).

For the practical energy and power densities of devices, the gravimetric and volumetric power ($P_m$, in W kg$^{-1}$, and $P_v$, in W cm$^{-3}$) and energy densities ($W_m$, in Wh kg$^{-1}$, and $W_V$, in Wh cm$^{-3}$) were calculated according to Supplementary Equations (2) and (3), respectively,

$$P_V = \int_{E_1}^{E_2} i(E)dE/(2V),\ P_m = \int_{E_1}^{E_2} i(E)dE/(2m) \quad (2)$$

$$W_V = \Delta E\int_{E_1}^{E_2} i(E)dE/(7200vV),\ W_m = \Delta E\int_{E_1}^{E_2} i(E)dE/(7200vm). \quad (3)$$

For the theoretical specific capacitance of $VO_2$, the theoretically specific capacitance ($C_{st}$) of $VO_2$ is evaluated according to Supplementary Equation (4),

$$C_{st} = nF/(ME) \quad (4)$$

where $n$ is the mean number of electrons transferred in the redox reaction, $F$ = 96485.34 C mol$^{-1}$ is the Faraday constant, $M$ is the molar mass of $VO_2$.

**Supplementary Note 2.** EIS analysis. For the EIS spectra of NP $c$-$V_2O_3$/$r$-$VO_{2-x}$, $c$-$V_2O_3$ and $r$-$VO_2$, they display characteristic semicircles with different diameters in the high- to middle-frequency range, followed by inclined lines with different slopes in the low frequency. Based on the equivalent circuit with general descriptors: At high frequency, the intersection point on the real axis represents the intrinsic resistance of

electrode and electrolyte ($R_I$); the semicircle behavior in the high- to middle-frequency range corresponds to the parallel connection of the interfacial charge transfer resistance ($R_{CT}$) and the EDLC ($C_F$); and the inclined line at low frequencies is attributed to ion diffusion inside the electrode with the slope denoting the Warburg resistance ($Z_w$), their values of $R_I$, $R_{CT}$ and $Z_w$ are estimated by using complex nonlinear least-squares fitting method.

## Supplementary References


1. Lin, T., Chen, I.W., Liu, F., Yang, C., Bi, H., Xu, F. & Huang, F. Nitrogen-doped mesoporous carbon of extraordinary capacitance for electrochemical energy storage. *Science* **350**, 1508–1513 (2015).
2. Li, Q., Wang, Z.L., Li, G.R., Gao, R., Ding, L.X. & Tong, Y.X. Design and synthesis of $MnO_2$/Mn/$MnO_2$ sandwich-structured nanotube arrays with high supercapacitive performance for electrochemical energy storage. *Nano Lett.* **12**, 3803–3807 (2012).
3. Choi, B.G., Yang, M., Hong, W.H., Choi, J.W. & Huh, Y.S. 3D mcaroporous graphene frameworks for supercapacitors with high energy and power densities. *ACS Nano* **6**, 4020–4028 (2012).
4. Chen, Z., Augstyn, V., Wen, J., Zhang, Y., Shen, M., Dunn, B. & Lu, Y. High-performance supercapacitors based on interwined CNT/$V_2O_5$ nanowire nanocomposites. *Adv. Mater.* **23**, 791–795 (2011).
5. Ghosh, A., Ra, E.J., Jin, M., Jeong, H.K., Kim, T.H., Biseas, C. & Lee, Y.H. High pseudocapacitance from ultrathin $V_2O_5$ films electrodeposited on self-standing carbon-nanofiber paper. *Adv. Funct. Mater.* **21**, 2541–2547 (2011).
6. Pech, D., Brunet, M., Durou, H., Huang, P., Mochalin, V., Gogotsi, Y., Taberna, P.L., Simon, P. Ultrahigh-power micrometer-sized supercapacitors based on onion-like carbon. *Nat. Nanotechnol.* **5**, 651–654 (2011).
7. El-Kady, M.F., Ihns, M., Li, M.P., Hwang, J.Y., Mousavi, M.F., Chaney, L., Lech, A.T. & Kaner, R.B. Engineering three-dimensional hybrid supercapacitors and microsupercapacitors for high-performance integrated energy storage. *Proc. Natl. Acad. Sci. USA* **112**, 4233–4238 (2015).
8. El-Kady, M. F. & Kaner, R. B. Scalable fabrication of high-power graphene micro-supercapacitors for flexible and on-chip energy storage. *Nat. Commun.* **4**, 1475 (2013).
9. El-Kady, M.F., Strong, V., Dubin, S. & Kaner, R.B. Laser scribing of high-performance and flexible graphene-based electrochemical capacitors. *Science* **335**, 1326–1330 (2012).
